# Evaluation of the coverage and depth of transcriptome by RNA-Seq in chickens

**DOI:** 10.1186/1471-2105-12-S10-S5

**Published:** 2011-10-18

**Authors:** Ying Wang, Noushin Ghaffari, Charles D Johnson, Ulisses M Braga-Neto, Hui Wang, Rui Chen, Huaijun Zhou

**Affiliations:** 1 Department of Poultry Science, Texas A &M University College Station, TX 77843-2472, USA; 2 AgriLife Genomics and Bioinformatics Services, Texas A&M University, College Station, TX 77843-2312, USA; 3 Department of Electrical and Computer Engineering, Texas A&M University, College Station, TX 77843, USA; 4 Department of Molecular and Human Genetics, Baylor College of Medicine, Houston, TX 77030, USA

## Abstract

**Background:**

RNA-Seq is the recently developed high-throughput sequencing technology for profiling the entire transcriptome in any organism. It has several major advantages over current hybridization-based approach such as microarrays. However, the cost per sample by RNA-Seq is still prohibitive for most laboratories. With continued improvement in sequence output, it would be cost-effective if multiple samples are multiplexed and sequenced in a single lane with sufficient transcriptome coverage. The objective of this analysis is to evaluate what sequencing depth might be sufficient to interrogate gene expression profiling in the chicken by RNA-Seq.

**Results:**

Two cDNA libraries from chicken lungs were sequenced initially, and 4.9 million (M) and 1.6 M (60 bp) reads were generated, respectively. With significant improvements in sequencing technology, two technical replicate cDNA libraries were re-sequenced. Totals of 29.6 M and 28.7 M (75 bp) reads were obtained with the two samples. More than 90% of annotated genes were detected in the data sets with 28.7-29.6 M reads, while only 68% of genes were detected in the data set with 1.6 M reads. The correlation coefficients of gene expression between technical replicates within the same sample were 0.9458 and 0.8442. To evaluate the appropriate depth needed for mRNA profiling, a random sampling method was used to generate different number of reads from each sample. There was a significant increase in correlation coefficients from a sequencing depth of 1.6 M to 10 M for all genes except highly abundant genes. No significant improvement was observed from the depth of 10 M to 20 M (75 bp) reads.

**Conclusion:**

The analysis from the current study demonstrated that 30 M (75 bp) reads is sufficient to detect all annotated genes in chicken lungs. Ten million (75 bp) reads could detect about 80% of annotated chicken genes, and RNA-Seq at this depth can serve as a replacement of microarray technology. Furthermore, the depth of sequencing had a significant impact on measuring gene expression of low abundant genes. Finally, the combination of experimental and simulation approaches is a powerful approach to address the relationship between the depth of sequencing and transcriptome coverage.

## Background

The transcriptome catalogues the complete set of transcripts in a cell. Transcriptomic regulation is critical to all physiological, developmental and pathological processes [1], and mRNA expression profiles can represent the characteristics of a cell at a specific state and help to govern its present and future activities [2]. The profiles of a transcriptome in terms of alterations in response to specific biological stimuli provides valuable insights for interpreting functional elements of the genome, revealing the molecular constituents of cells, and also understanding developmental and disease processes.

Different types of technologies have been developed to interrogate transcript abundance, including hybridization-based and sequencing-based approaches. Hybridization-based microarrays have been the primary transcriptomic high-throughput tool for almost two decades, which has accelerated the study of transcriptome analysis by profiling thousands of genes simultaneously [3]. However, microarray technology has several limitations including: indirect quantification by hybridization-signal intensities [4], background and cross-hybridization problems [5] and reproducibility issues [6]. The development of next generation sequencing with improved qualitative and quantitative measurements holds great promise in transcriptome analysis.

RNA-Seq is a recently developed approach to map and quantify transcriptomes by digitally recording how frequently each transcript is represented in a sequence sample. After poly (A) selection, RNA is fragmented to small fragments and converted into a cDNA library, which provides a simple and more comprehensive way to measure transcriptome composition and to discover new genes by high-throughput sequencing without bacterial cloning of cDNA input [2]. Studies using this technology have already altered our views regarding the extent and complexity of transcriptomes in an organism and dramatically improved our understanding of transcriptome. RNA-Seq has several advantages over micorarrays including: 1) RNA-Seq is not dependent on prior knowledge about the target sequence; 2) It has a large dynamic range and sensitivity due to its digital nature, which is especially important for highly abundant and extremely low abundant genes; 3) The survey of a transcriptome is more accurate because the quantification of each transcript is directly based on digital counts of the transcript. Therefore, RNA-Seq offers both single-base resolution for annotation and “digital” quantification at the RNA level, which allows the entire transcriptome to be analyzed in a high-throughput and quantitative manner [7]. However, the expense per sample for RNA- Seq is still a limiting factor in preventing researchers from sequencing multiple biological replicates per group, which are needed for statistically-significant analysis. It is common to adopt a pooling strategy to reduce the cost for RNA-Seq studies [8]. With the continued enhancement of sequencing output and the development of multiplex labelling techniques, the cost per sample could be significantly reduced if several samples are multiplexed and sequenced in the same lane, given sufficient transcriptome coverage per sample. Therefore, it is imperative to address the trade-off between the depth of RNA-Seq and the coverage of the transcriptome in an organism. The objective of this study was to evaluate what coverage or sequencing depth of transcriptome would be sufficient to interrogate gene expression profiling in the chicken by RNA-Seq.

## Methods

### RNA preparation

Total RNA was isolated from four chicken lungs from two genetic chicken lines (two samples per line) by Trizol (Invitrogen, Carlsbad, CA) according to the manufacturer’s protocol. Two RNA samples from the same line were pooled to generate totals of two pooled RNA samples (Sample1 and Sample2). DNase I (Ambion, Austin, TX) digestion was carried out after RNA isolation and the RNA concentration and purity were determined by measuring absorbance at 260 nm and A260/A280 ratio using a NanoDrop ND-1000 spectrophotometer (Nano-drop Technologies, Wilmington, DE). RNA samples were stored at -80 °C until further use.

### cDNA library preparation and sequencing by RNA-Seq

Total RNA (7 µg) was subjected to two rounds of hybridization to oligo (dT) beads (Invitrogen, Carlsbad, CA) to enrich mRNA. Ribosomal RNA contamination was evaluated by RNA pico chip using a BioAnalyzer (Agilent, Santa Clara, CA). The resulting mRNA was then used to prepare cDNA libraries using the RNA sequencing sample preparation kit (Illumina, San Diego, CA). Sample1 and Sample2 were sequenced by Illumina Genome Analyzer and then Genome Analyzer II, which generated four datasets: S1-R1, S2-R1, and S1-R2, S2-R2, respectively.

### Data filtering, mapping reads and identifying transcriptome contents

The sequences generated went through a filtering process first. Any reads that contained numerous interspersed Ns in their sequences, or had relatively short reads (<17 bp), were removed for the following analysis. Sequence reads obtained after quality control with filtering were analyzed using CLC Genomics Workbench 4 (CLC bio, Cambridge, MD). After mapping, the gene expression level was quantified by simply dividing the number of reads mapped to each gene by the size of its transcripts, commonly known as the number of reads per kilobase of exon per million mapped reads (RPKM) [2], for all 15,742 annotated chicken genes in the database. The gene expression level was then log_2_ transformed.

### Random sampling of S1-R2 and S2-R2

We have obtained RNA-Seq data in three different levels of depth: 1.6 M, 4.9 M, and about 30 M reads. Clearly, there was a big gap between 4.9 M and 30 M reads. In order to identify the appropriate depth of transcriptome per sample that is sufficient for whole genome transcriptome profiling, it is important to generate additional datasets at different levels of depth. It would be very costly to re-sequence each sample to generate RNA-Seq data at different levels of sequencing depth. Random sampling from current dataset might provide a cost-effective approach for this purpose. This procedure synthetically created samples from the originally sequenced samples. Thus, for samples S1-R2 and S2-R2 data sets, by drawing without replacement a fixed number of reads from the overall data set, we randomly selected 10 M, 15 M and finally 20 M reads. These random selections are repeated 4 times, resulting in total of 24 technical replicates with different transcriptome depth. Each one of the reads in the FASTQ format of the input files, which were used in the sampling, was selected equally likely. A program in Perl was written to serve this purpose. Then, the resulting replicate datasets were uploaded into CLC Genomics Workbench in the FASTQ format for the analysis individually. The correlation coefficients of gene expression (RPKMs) between replicates of each sequencing depth were calculated by JMP (SAS, Cary, NC). The average RPKMs of transcripts identified by each sequencing depth (10 M, 15 M and 20 M) were calculated to represent the gene expression values for further analysis.

### Correlation coefficients between different sequencing depths from the same sample

In order to evaluate how reliable the sequence data is at each level of sequencing depth, correlation coefficients between lower and high depth sequence data for each sample were calculated by JMP (SAS, Cary, NC). Any genes with no gene expression at either sequence dataset were excluded from the correlation coefficient computation. The depth of sequencing is highly correlated with the abundance of gene expression, so we divided genes into the four quartile groups based on expression levels for each dataset, from the bottom 25% (1^st^ quartile) to the top 25% (4^th^ quartile). Subsequently, correlation coefficients between lower-depth sequence data and the data with 28.7- 29.6 M reads within each of the four quartile groups were calculated.

## Results

### RNA-Seq for cDNA libraries

The two chicken cDNA libraries (Sample1 and Sample2) were sequenced by the Illumina Genome Analyzer, which generated 4.9 M (60 bp) reads (S1-R1) and 1.6 M (60 bp) reads (S2-R1), respectively. Then, two technical replicate cDNA libraries from the same RNA samples were re-sequenced using the Genome Analyzer II, which generated 29.6 M (75 bp) reads (S1-R2) and 28.7 M (75 bp) reads (S2-R2).

### Random sampling of the S1-R2 and S2-R2

The datasets of S1-R2 (29.6 M) and S2-R2 (28.7 M) were each randomly re-sampled into 10 M, 15 M, and 20 M reads with four replicates each. The correlation coefficients between every two replicates for each re-sampled level (10 M, 15 M and 20 M) within each sample (S1-R2 and S2-R2) were all greater than 0.98, which demonstrated that the sampling procedure is consistent and accurate. Averages of gene expression from the four replicates at each re-sampled level for each sample were used for further analysis.

### Effects of sequence depth on the distributions of transcripts

**Figure 1:**
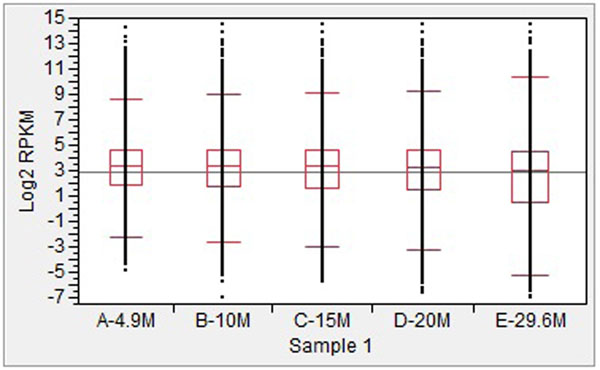
Distributions of log2 transformed reads of transcripts at different sequencing depths for Sample1.

**Figure 2:**
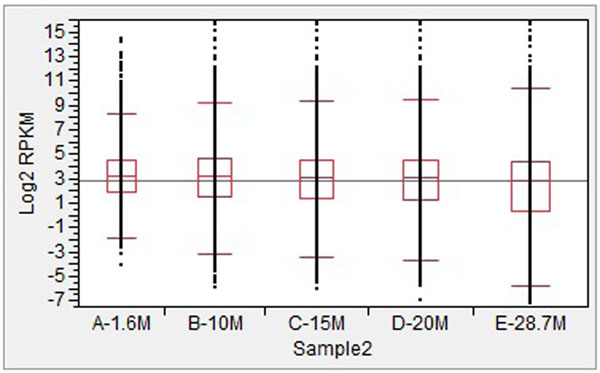
Distributions of log2 transformed reads of transcripts at different sequencing depths for Sample2.

The distributions of transcript abundance at different levels of sequence depth from Sample1 and Sample2 are presented in Figures 1 and 2, respectively. In general, the median and 75% percentile were similar across five different levels of depth, while the 95% percentile showed a slight increase, especially from 20 M to 30 M. Significant decreases at the 25% and 5% percentile were observed, especially from 20 M to 30 M. In addition, a significant decrease was also observed from 1.6 M to 10 M in Sample2.

### Coverage of annotated chicken genes

**Figure 3:**
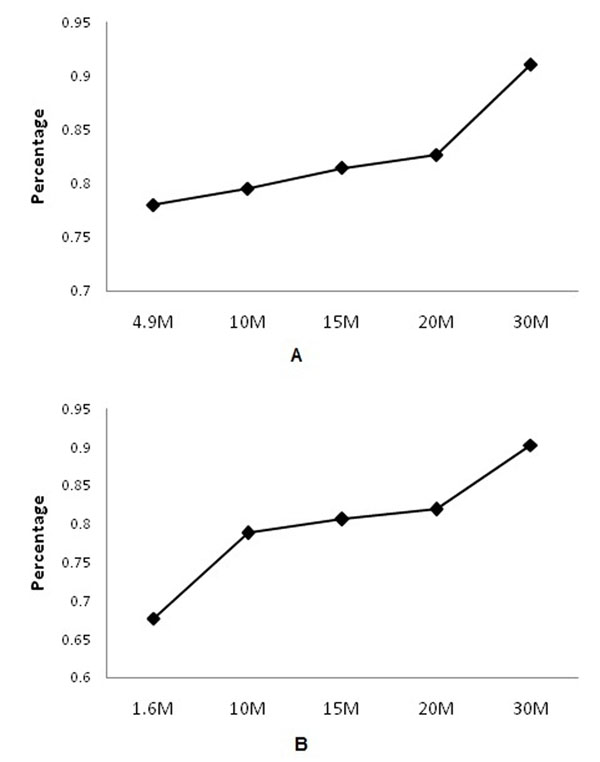
Percentages of detected chicken genes at different levels of sequence depth across all annotated chicken genes. A: Sample1; B: Sample2.

There are about 15,742 annotated chicken genes in the NCBI database. Number of detected chicken genes at different levels of sequencing depth from Samples 1 and 2 are presented in Table 1. There were 14,336 genes detected in S1-R2 (29.6 M) and 14,212 genes in S2-R2 (28.7 M), which accounted for 91.07% and 90.28% of all 15,742 annotated chicken genes in the database, respectively. With the reduction of sequencing depth, the number of detectable genes also decreased from 91% to 78% in Sample1 (Fig. 3A), and from 90% to 68% in Sample2 (Fig. 3B). Two significant drops of transcriptome coverage were observed: from 30 M to 20 M, and 10 M to 1.6 M.

**Table 1:** Numbers of detected annotated chicken genes at different levels of sequence depth.

***Sample1***		***Sample2***	
	***Numbers of genes***		******Numbers of genes******

**29.6 M**	14,336	**28.7 M**	14,212
**20 M**	13,011	**20 M**	12,895
**15 M**	12,822	**15 M**	12,690
**10 M**	12,515	**10 M**	12,406
**4.9 M**	12,276	**1.6 M**	10,664

### Correlation coefficients between different sequencing depths

**Figure 4:**
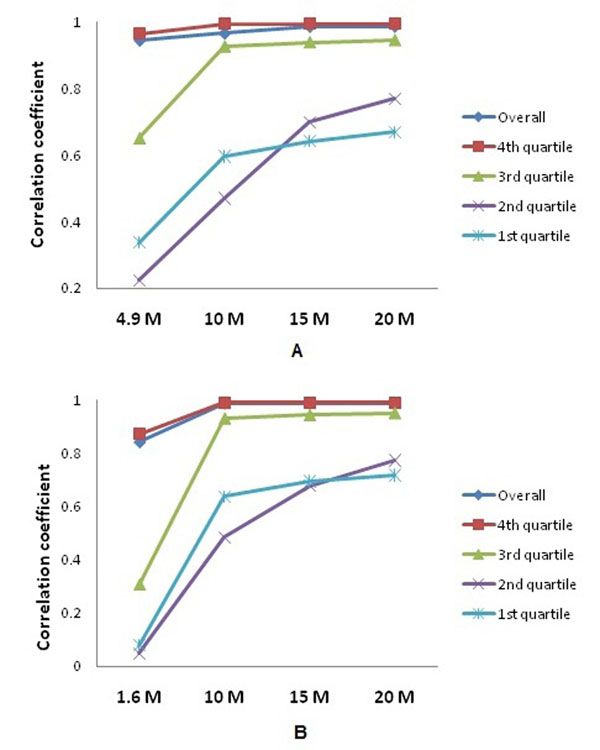
Correlation coefficients between 30 M reads and four different levels of sequence depth at different quartiles. A: Sample1; B: Sample2.

To evaluate the appropriate depth of sequence that is needed for transcriptome profiling, correlation coefficients between different levels of sequencing depth and the most abundant reads for each sample were calculated. For Sample1, overall correlation coefficients at four different levels of depth were greater than 0.95. When we examined the four quartile groups based on expression level (Fig. 4A), correlation coefficients ranged from 0.34 to 0.67 for the 1^st^ quartile, 0.22 to 0.77 for the 2^nd^ quartile, 0.65 to 0.95 for the 3^rd^ quartile, and 0.97 to 1.0 for the 4^th^ quartile. A similar pattern in terms of correlation coefficient change was observed for the 1^st^, 2^nd^, and 3^rd^ quartiles; a significantly increased correlation coefficient from 4.9 M to 10 M, and kept relative flat from 10 M to 20 M. For the 4^th^ quartile, correlation coefficients at four different levels of depth were greater than 0.95. From the 1^st^ to the 4^th^ quartiles, there were significant increases for correlation coefficients between every two quartile groups (*p*<0.01). For Sample2, overall correlation coefficients at four different levels of depth were greater than 0.98, except for 1.6 M at 0.84. Correlation coefficients ranged from 0.08 to 0.72 for the 1^st^ quartile, 0.05 to 0.78 for the 2^nd^ quartile, 0.31 to 0.95 for the 3^rd^ quartile, and 0.87 to 1.0 for the 4^th^ quartile (Fig. 4B). The same pattern in terms of correlation coefficients changes at different levels of depth between Sample1 and Sample2 was also observed.

## Discussion

In the current study, RNA-Seq was performed twice using two chicken cDNA samples. The first run of RNA-Seq had fewer number of reads and larger variation in terms of total number of reads between the two samples, while the second run had greater number of reads and very small variation between the two samples. The first run was performed at the very early stage of the sequencing technology when it was still in the testing phase. The lower reads and larger variation in the first run may be coming from two major sources of technical error: the purification of cDNA templates during the library preparation, and the loading of libraries onto flow cells (RNA-Seq technical guide and personal communications, Illumina technical support staff). These potential sources of errors were corrected during the second RNA-Seq analysis, which provided very good sequencing depth with greater number of reads. The first RNA-Seq datasets were directly derived from actual experiment, which made the results more informative than replicating datasets by random sampling. Therefore, we chose to include these two early datasets in the analysis in the current study. Furthermore, all of the reads from each sample were normalized by the RPKM and the datasets can serve as a reference for random sampling at different sequencing depths from the exact same samples.

The capacity of sequencing length of 60 bp for the first run was increased to 75 bp at the second RNA-Seq analysis. Longer reads should reduce estimation error and mapping uncertainty, and read lengths have been consistently increasing with improving Illumina massively parallel sequencing technology. However, people have noted that the number of reads is more important than the read length once reaching a minimum read length of 25 bp [9,10]. The read lengths (60 bp and 75 bp) in the current study were larger than 25 bp; therefore, the read length will not affect the overall conclusions drawn.

As a powerful new technology for transcriptome analysis, RNA-Seq provides a more comprehensive view of the transcriptome than earlier technologies. Besides its ability to detect splicing variation, RNA editing and discovery of new transcripts [11], RNA-Seq can also function in the role of a conventional microarray in measuring gene expression due to its accurate measurements. In order to detect less abundant transcripts, appropriate sequencing depth is needed. The transcriptome coverage or sequencing depth needed for a given study can be affected by several factors such as genome size, transcriptome complexity and objectives of the study. In general, the more complex the transcriptome, the more sequencing depth is required for adequate coverage [12]. Depending on the purpose of transcriptome analysis, the requirement of sequencing depth varies. In most transcriptome studies, quantifying mRNA abundance is one of the major objectives. There is a certain sequencing depth that is sufficient in simple transcriptomes. For example, in the yeast genome, a 29.9 M (35 bp) reads dataset was generated by RNA-Seq which was able to get 100% transcriptome coverage [13]. The number of transcripts detected by RNA-Seq in the yeast dataset was able to reach 80% transcriptome coverage at 4M mapped reads, and even though the sequencing depth doubled as 8M reads, the transcriptome coverage only increased 10% [13,14]. These results suggest that the improvement of sequencing depth or transcriptome coverage after reaching a certain sequencing depth had relatively less impact on detecting low abundant genes [15]. In addition, the cost per sample per lane by RNA-Seq is still not affordable for most laboratories. Recent development in multiplex labelling using bar-coded libraries by Illumina and continued increase in sequence output have made it possible to sequence multiple samples per lane without extra cost or running time [16]. Therefore, it is imperative to examine the correlation between sequencing depth and transcriptome coverage; in other words, what sequencing depth might be sufficient in reaching a certain level of transcriptome coverage and reliable measurement for RNA-Seq. In order to accomplish this objective, two approaches could be applied: experimental or simulation methods. Both methods have been applied in this study. High correlation among replicates within each sequencing depth, gradual increase in correlation coefficients from 10 M to 20 M, and consistent patterns observed between Samples1 and 2 (Fig. 4) have demonstrated that random sampling was an effective and reliable method in reaching the goals of this study.

Transcriptome coverage is one of the most important parameters in profiling global gene expression. The number and level of transcript isoforms is not always known and transcription activity varies across the genome [14]. This was confirmed in a study by using the number of unique transcription start sites as a measure of coverage in mouse cells [15]. In the current study, we took a more practical approach using all annotated genes in the chicken genome. Because the chicken genome is far under-annotated, we assume that the 15,742 annotated chicken genes in the database would well represent different levels of expression abundance in the chicken genome, which is essential for the analysis of transcriptome coverage in this study. Since gene expression depends on tissue and time of biological process [15], it is impossible for any single tissue to have all genes in the genome expressed. Ninety percent of all annotated genes (Fig. 3) detected at about 30 M (75 bp) reads might represent a saturated detection of the whole genome. The analysis results indicated significant improvements of transcriptome coverage occurred from 1.6 M to 4.9 M and from 20 M to 30 M. Depending on the purpose of transcriptome analysis, the current study suggested that 10 M (75 bp) reads could have 80% of transcriptome coverage, while 30 M (75 bp) reads could reach 90% of transcriptome coverage.

When we analyzed overall correlation coefficients at different levels of sequencing depth regardless of gene expression level, we observed very high correlation coefficients between each level of sequencing depth compared with 30 M, except for 1.6 M. One might draw a conclusion that there is no significant difference among different levels of sequencing depth. But as we see in Figure 4, this might be true in the case of highly abundant genes (the 4^th^ quartile group), but not in the case of the 1^st^ to 3^rd^ quartile groups, especially the first two quartile groups (i.e. expression below the median). High abundant genes will be less affected by sequencing depth than low abundant genes, because high abundant genes are more likely to be captured even when the sequencing depth varies [17]. This is also confirmed by our analysis. Collectively, the following points can be inferred: 1) Sequencing depth below 20 M (75 bp) reads had a significant effect on detecting transcript levels of medium and low abundant transcripts; 2) Sequencing depth at both 1.6 M and 4.9 M would result in unreliable mRNA expression on all genes except highly abundant transcripts; 3) There was no significant improvement in correlation coefficients when sequencing depth doubled from 10 M to 20 M. Based on these analysis, the results suggested: 1) 5 M reads might be sufficient in obtaining reliable mRNA expression measurement on highly abundant transcripts; 2) When sequencing depth is beyond 10 M reads, a relatively reliable measurement of mRNA expression is expected, especially for abundant transcripts; 3) It seems that 30 M of reads is needed to achieve reliable measurement of mRNA expression across all genes in the chicken genome. To our knowledge, this is the first study evaluating the appropriate sequencing depth using RNA-Seq in farm animals and will provide the first reference for similar studies. The knowledge generated from this study has laid a solid foundation for applying this analysis to other species.

## Conclusions

In summary, the analysis from this study demonstrated that 30 M (75 bp) reads is sufficient to detect all annotated genes and provide a reliable measurement of mRNA abundance in chicken lungs using RNA-Seq. As we expected, the depth of sequencing had significant impact on low abundance transcripts, but not on high abundance transcripts. In practice, if RNA-Seq serves as a replacement of microarray technology, 10 M (75 bp) reads would allow detection of about 80% of annotated chicken genes. Finally, increasing the depth of sequencing from 10 M to 20 M reads did not have a significant effect on transcriptome coverage and reliability of mRNA measurements, whereas 30 M reads was needed to achieve reliable measurement of mRNA expression across all genes in the genome.

## Competing interests

The authors declare that there are no competing interests.

## Authors' contributions

YW analyzed the data and drafted the manuscript, NG wrote the code for sampling the RNA-Seq data, CJ and UB contributed ideas for the data analysis and revised the manuscript, HW and RC ran RNA-Seq samples, and HZ designed the experiment, provided the concept of the analysis. All authors submitted comments, read and approved the final manuscript.
